# Calcium Reduces Fruit Abscission in *Persimmon* by Targeting Cell Wall Integrity

**DOI:** 10.3390/plants14223482

**Published:** 2025-11-14

**Authors:** Andrés Marzal, Julia Morales, Amparo Primo-Capella, Almudena Bermejo, Amparo Martínez-Fuentes, Ana Quiñones

**Affiliations:** 1 Centro para el Desarrollo de la Agricultura Sostenible, Instituto Valenciano de Investigaciones Agrarias, 46113 Moncada, Valencia, Spain; morales_jul@gva.es (J.M.);; 2 Centro de Citricultura y Producción Vegetal, Instituto Valenciano de Investigaciones Agrarias, 46113 Moncada, Valencia, Spain; 3 Instituto Agroforestal Mediterráneo, Universitat Politècnica de València, 46022 Valencia, Valencia, Spain

**Keywords:** fertilisation management, persimmon, abscission zone, gene expression, polygalacturonase

## Abstract

In the Mediterranean region, the persimmon cultivar ‘Rojo Brillante’ may experience up to four waves of fruit drop. The first is a physiological event during fruit set that is common in woody species, while the subsequent waves are induced by rising temperatures and prolonged summer water stress. These summer drops represent the main limiting factor, leading to yield losses of up to 90%. Organ abscission is a complex process regulated by genetic, hormonal, nutritional, and environmental factors. We hypothesise that calcium (Ca) plays a protective role in the abscission zone (AZ) by inhibiting cell wall-degrading enzymes such as polygalacturonase (PG) and pectin methylesterases (PMEs). Calcium applications every 15 days from anthesis onwards significantly reduced fruit drop. Treatments preserved polar auxin transport—through *DkPIN1* expression—and inhibited stage C of the abscission process, decreasing the relative expression of the *DkIDL6* gene in the AZ. Moreover, PME and PG activities were significantly lower in Ca-treated fruits, confirming the stabilising effect of calcium on AZ integrity. In summary, pre-anthesis calcium sprays reduced premature fruit drop by about 30% under heat–drought stress by down-regulating key abscission genes (*DkIDL6*, *DkPG20*, *DkPME41*) and preserving cell wall integrity and fruit firmness, supporting the use of Ca treatments as a climate-smart approach to stabilise persimmon yield.

## 1. Introduction

In fruit tree crops, premature abscission represents/is one of the main physiological factors limiting yield in citrus (*Citrus* spp.) [1], apple (*Malus domestica* Borkh.) [2], and avocado (*Persea americana* Mill.) [3]. Recently, this problem has also emerged in persimmon (*Diospyros kaki* Thunb), where up to four waves of fruit abscission can occur [4].

Abscission is a cell separation process through which a plant sheds its aerial organs. This occurs in a thin layer of specialised cells known as the abscission zone (AZ), which separates the organ from the rest of the plant [5,6,7]. This process plays a key role in plant evolutionary adaptation by promoting seed dissemination, discarding senescent tissues, and removing damaged organs. The model plant *Arabidopsis thaliana* has been used to study the mechanisms of fruit abscission [8]. The process consists of four stages [9]: (i) the determination of the AZ (stage A), (ii) the acquisition of the competence to respond to abscission signals (stage B), (iii) the activation of abscission allowing organ separation (stage C), and (iv) the differentiation of a shielding layer (stage D). In this model, auxin plays a key protective role. During stage B, the depletion of the basipetal auxin flux in the AZ renders the tissue sensitive to ethylene, which then triggers abscission, through the activation of cell-wall-degrading enzymes such as polygalacturonase (PG) or pectin-methylesterase [10,11,12]. The question arises as to whether this model is valid for all types of species, particularly more complex ones such as woody plants. In tree species, fruits often persist for extended periods and may detach from multiple locations [13] (at different phenological stages [14]). According to [15], advancing the understanding of abscission requires the application of fundamental knowledge derived from this model to more complex scenarios, such as these types of crops.

Abscission has been extensively studied in citrus, particularly due to the high sensitivity of navel orange cultivars to this phenomenon [16]. The main agronomic practice in citrus-producing regions is the application of the synthetic auxin 2,4-dichlorophenoxyacetic acid (2,4-D) to prevent fruit drop and maintain yield [17,18]. However, in persimmon, studies have focused on fruit set [19,20] rather than on premature abscission during stage II of fruit development. Consequently, the molecular mechanism of abscission in persimmon has not been described, nor have any agronomic strategies been developed to manage it. Therefore, a possible solution could be the use of synthetic auxins, as in citrus; however, persimmon is highly sensitive to these treatments, and their use is increasingly restricted by the European Union. A thorough understanding of the mechanisms underlying fruit abscission in persimmon is essential to develop new and effective cultural practices.

Histological studies of persimmon have identified two main detachment zones: one in the pedicel (AZ-A) and another in the calyx (AZ-C) [20,21,22]. Early abscission of flowers and fruitlets occurs through AZ-A, which becomes progressively inactivated towards the end of stage I of fruit development, whereas abscission at AZ-C takes place during the subsequent waves of summer fruit drop. While all agronomic techniques have focused on applying hormonal treatments to protect the AZ at stage B [17,18,23], intervention at stage C, during cell wall degradation caused by enzymes, is not extensively studied. The integrity of the abscission zone depends largely on the cohesion of the cell wall, which is primarily maintained by pectic substances in association with calcium ions [24,25]. In fact, the activity of the pectin-hydrolysing enzyme polygalacturonase (PG) in ripe grape berries was inhibited in vitro by calcium concentrations of 1 mM or higher in [26]. Furthermore, in tomatoes (*Solanum lycopersicum* L.), the application of calcium-chelating agents such as EDTA or citrate reduces resistance to PG attack by solubilising calcium ions [27].

In addition to reinforcing cell wall cohesion, free Ca^2+^ ions function as crucial secondary messengers in numerous plant adaptive processes [28], suggesting their potential as a strategy to control premature fruit drop. These responses are mediated by distinct families of Ca^2+^ sensors, including calmodulins (CaMs), Ca^2+^-dependent protein kinases (CDPKs), calcineurin B-like proteins (CBLs), and their interacting protein kinases (CIPKs) [29]. Notably, an increase in calcium fraction I (free Ca^2+^ ions) in the abscission zone (AZ) is associated with abscission, as high concentrations of Ca^2+^ activate transmembrane ion channel receptors that trigger a protein kinase cascade regulating the transcription and translation of specific proteins to achieve the intended physiological response [10]. Indeed, in tomato, elevated cytosolic Ca^2+^ levels stabilise the calcium-dependent protein kinase CPK10, which in turn activates the expression of the gene *IDL6* [30], a key regulator of AZ degradation at stage C, involved in pectin breakdown and cell wall remodelling [31]. However, when Ca^2+^ moves into other calcium fractions, such as calcium pectate and calcium carbonate, both fundamental components of the cell wall and membrane [32], cellular protection is enhanced by limiting the activity of pectin-hydrolysing enzymes, thereby increasing cell wall hardening [33]. These observations highlight the potential of calcium treatments as a strategy to reduce premature fruit drop in persimmon.

The physiological and molecular mechanisms underlying persimmon fruit abscission remain to be elucidated. Preventing abscission using alternative or complementary agronomic techniques to hormonal treatments is, therefore, of interest. This study hypothesises that sequential calcium application in young persimmon fruits delays abscission by inhibiting the activity of pectin-hydrolysing enzymes and enhancing cell wall integrity, thereby preventing cell wall dissolution during abscission stage C. Therefore, this study focused on identifying the physiological and molecular processes involved in persimmon fruit abscission. It also evaluated whether sequential calcium treatments could limit premature fruit drop by maintaining abscission zone integrity.

## 2. Results

### 2.1. Fruit Drop

**Figure 1:**
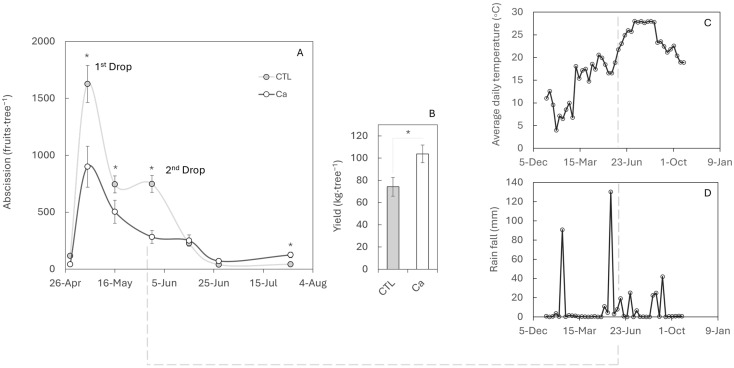
Effect of calcium applications on fruit set and premature fruit drop. (**A**) Number of dropped fruits recorded every 15 days using nets placed under control (CTL) and calcium-treated (Ca) trees. (**B**) Yield at harvest in October. Values represent the mean of three replicates of five trees each. Asterisks indicate significant differences (*p* ≤ 0.05). Standard errors (SEs) are indicated by vertical bars; in some cases, the SE is smaller than the symbol. (**C**) Average daily temperature and (**D**) rainfall were monitored throughout the productive period.

Fruit drop intensity during fruit set is linked to the number of ovaries that initiate development. In persimmon, abundant flowering causes a strong first drop, which in control trees (CTL) reached up to 45% of total fruits dropped during the whole studied period. This first physiological fruit drop is common in woody species to regulate fruit load. However, a second drop occurred in this study year at the onset of exponential fruit growth (Figure 1A), and this stage (together with the third or fourth drop, depending on the year) currently represents one of the main limitations to production. Calcium (Ca) application reduced fruit losses during the first drop by ~55% with the control fruit and completely prevented the second peak drop (Figure 1A) compared with control trees, resulting in an average yield at harvest that was ~30% higher than that of the control trees (Figure 1B). In addition, Ca treatment improved fruit weight, height, and firmness and delayed the change of colour, which could extend the harvest period (Table 1).

Environmental factors are key in triggering premature fruit abscission. The second fruit drop correlates with rising average temperatures in June (Figure 1C) and with a prolonged period of water stress interrupted by heavy rainfall of 130 mm (Figure 1D). However, abscission is a complex process that also involves additional endogenous factors, such as hormonal, genetic, and nutritional components. Thus, while the occurrence of a second, third, or even fourth wave of fruit drop is highly variable and depends on summer climatic conditions and the physiological state of the plant, only the first and second waves were analysed in this study.

### 2.2. Molecular Signature of Fruit Abscission in Persimmon

**Figure 2:**
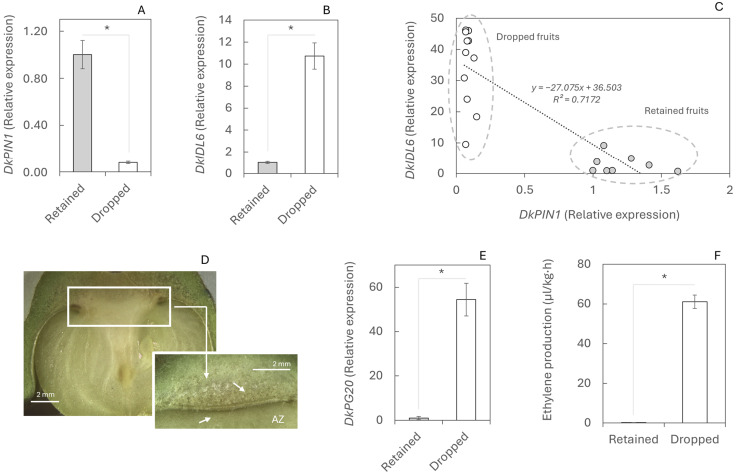
Abscission mechanism during physiological fruit drop in retained and dropped fruits. Gene expression of *DkPIN1* (**A**), *DkIDL6* (**B**), and *DkPG20* (**E**)—involved in polar auxin transport, initiation of the abscission process (stage C), and cell wall pectin degradation, respectively—was determined by RT-qPCR. (**C**) Correlation between *DkPIN1* and *DkIDL6*. (**D**) Calyx abscission zone. (**F**) Ethylene production in fruits abscised during the night. Twelve fruits were analysed; for RT-qPCR, each value corresponds to a single fruit, whereas three biological replicates of three fruits each were used for ethylene measurements. Asterisks indicate statistically significant differences (*p* ≤ 0.05). Standard errors (SEs) are shown as vertical bars.

In order to analyse the molecular mechanisms underlying fruit abscission that consistently occurs as the first drop of the season in persimmon, the relationship between auxin flux (*DkPIN1*) and the abscission-triggering gene *DkIDL6* was evaluated during this event. The expression of the *DkPIN1* gene in retained fruits was 12-fold higher than in dropped fruits (Figure 2A), indicating a continuous auxin flux. Conversely, *DkIDL6* expression in dropped fruits was 10-fold higher than in retained fruits (Figure 2B). A large-scale analysis of numerous fruits revealed a clear correlation [*y* = *x* + *b*] between these two genes (*r* = −0.847; *p*-value < 0.001), showing that dropped fruits exhibit higher *DkIDL6* and lower *DkPIN1* expression compared to retained fruits, effectively separating the two populations (Figure 2C).

Overexpression of *DkIDL6* in dropped fruits activates the genetic program for cell degradation in the abscission zone (AZ; Figure 2D). The expression of the *DkPG20* gene, which encodes a polygalacturonase involved in cell wall breakdown, was 60-fold higher in the AZ of dropped fruits than in retained fruits (Figure 2E). Furthermore, another trigger of abscission, ethylene, was detected at elevated levels in dropped fruits. Ethylene production in these fruits was 61.08 µL·kg^−1^·h^−1^, compared to only 0.98 µL·kg^−1^·h^−1^ in retained fruits (Figure 2F).

**Figure 3:**
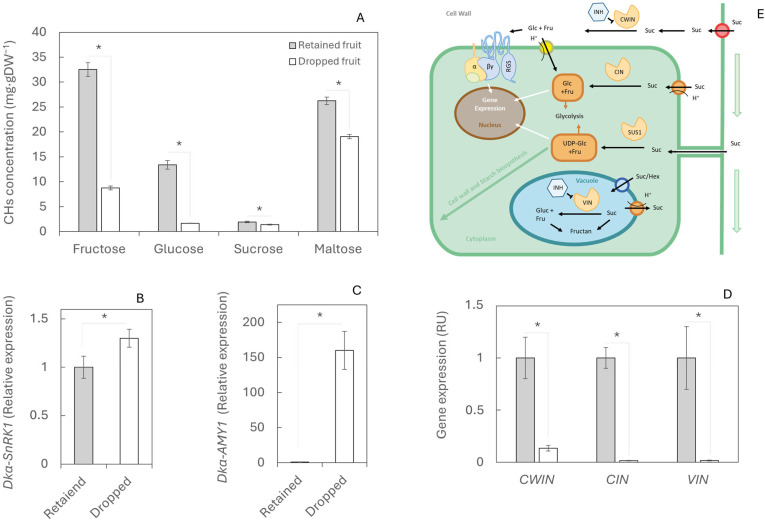
Carbohydrate content and metabolism during physiological fruit drop in retained and dropped fruits. (**A**) Concentrations of the main sugars (fructose, glucose, sucrose, and maltose) measured in 20 retained and 20 dropped fruits. (**B**) Gene expression of *Dkα-SnRK1*, (**C**) *Dkα-AMY1*, and (**D**) invertases (*DkCWIN*, *DkCIN*, *DkVIN*)—involved in sugar starvation signalling, starch hydrolysis, and sucrose metabolism, respectively—was determined by RT-qPCR. Twelve fruits per treatment were analysed; for gene expression, each value corresponds to a single fruit. (**E**) Schematic representation of sugar metabolism. Asterisks indicate statistically significant differences (*p* ≤ 0.05). Standard errors (SEs) are shown as vertical bars.

Carbohydrates also play a crucial role in fruit development. Concentrations of fructose, glucose, sucrose, and maltose were higher in retained fruits than in dropped fruits (Figure 3A), with the largest differences observed in glucose and fructose, which were 87.6% (32.51 mg gDW^−1^) and 73% (13.39 mg gDW^−1^) higher, respectively, in retained fruits (Figure 3A). This carbohydrate shortage in dropped fruits was associated with a strong increase in the expression of the gene *Dkα-SnRK1* (Figure 3B), indicating nutritional stress. As a result, starch biosynthesis appeared to be activated, as reflected by the overexpression of *Dkα-AMY1*, reaching more than 150 relative units in dropped fruits compared with retained fruits (Figure 3C). By contrast, the expression of the invertase genes *CWIN*, *CIN*, and *VIN*, which hydrolyse sucrose into glucose and fructose, remained more than nine-fold lower in dropped fruits than in retained fruits (Figure 3D).

### 2.3. Calcium Effect on Second Drop Abscission

**Figure 4:**
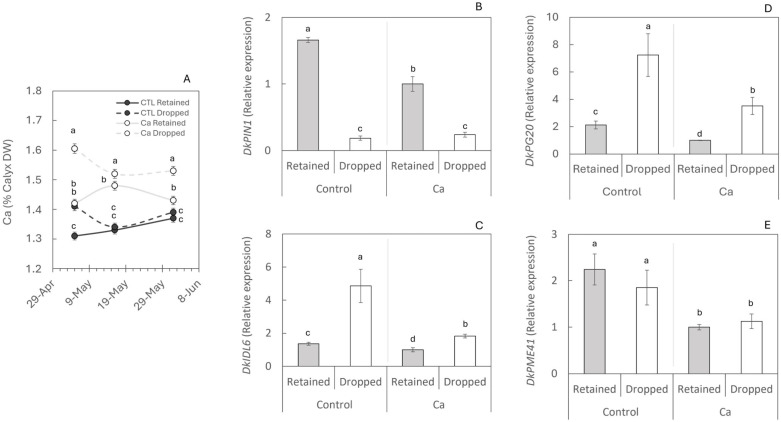
Effect of calcium treatments on the molecular mechanism of abscission. (**A**) Calcium concentration in the calyx of 20 retained and 20 dropped fruits per treatment. Gene expression of *DkPIN1* (**B**), *DkIDL6* (**C**), *DkPG20* (**D**), and *DkPME41* (**E**)—involved in polar auxin transport (**B**), initiation of the abscission process at stage C (**C**), and cell wall pectin degradation (**D**,**E**)—was determined by RT-qPCR. Five fruits per treatment were analysed, and each value represents a single fruit. Letters indicate statistically significant differences (*p* < 0.05). Standard errors (SEs) are shown as vertical bars.

Having identified the molecular mechanism of persimmon fruit abscission, the effect of Ca on preventing abscission during the second wave was analysed. The concentration of Ca in the calyx increased rapidly (15 days after the first treatment). Calyx Ca concentration increased 15 days after the first application, by 8.4% in retained fruits and 13.1% in dropped fruits, and these differences remained for the rest of the period (Figure 4A).

At this second wave, control trees showed the same behaviour as observed during the first drop (Figure 4). In abscised fruits, the expression of the auxin efflux carrier *DkPIN1* was significantly reduced (Figure 4A), leading to abscission through *DkIDL6* gene expression (Figure 4B) and subsequent cell wall degradation via up-regulation of *DkPG20* (Figure 4C). Calcium application modulated the expression of the four studied genes, generally lowering transcript levels relative to untreated controls (Figure 4B–E). For *DkPIN1*, Ca reduced expression in retained fruits but did not change it in dropped fruits (Figure 4B). By contrast, *DkIDL6* and *DkPG20* decreased in both retained and dropped fruits under Ca treatment; notably, in dropped fruits, the Ca treatment also reduced data dispersion, indicating lower between-fruit variability (Figure 4C,D). In control fruits, *DkPME41* expression did not differ between retained and dropped fruits, and a similar pattern was observed in Ca-treated fruits. Calcium treatment nonetheless reduced *DkPME41* expression in both categories (Figure 4E). Overall, these patterns indicate that Ca attenuated the abscission program across fruit categories, with *DkPIN1* unchanged in dropped fruits and a uniform down-regulation of *DkIDL6*, *DkPG20*, and *DkPME41*.

## 3. Discussion

Calcium functions as an inhibitor of fruit and leaf abscission in woody plants such as citrus [34,35]. The role of calcium in delaying abscission was recognised nearly a century ago [33]. However, the precise mechanism by which calcium prevents abscission remains unclear. This is partly due to the species-specific responses to calcium. For instance, while calcium application reduces pedicel abscission in tomato explants [36], it has no significant effect in apple trees [37]. Besides this, the effectiveness of calcium treatments also depends on both the concentration and the timing of application, owing to its low mobility within the plant. Under Mediterranean summers, prolonged heat waves and scarce rainfall produce strong abiotic stress in persimmon orchards, reducing growth and predisposing trees to premature fruit drop. Recent field evidence comparing Spain vs. Japan showed greater than 35 °C episodes and markedly lower precipitation at the Spanish site, with transcriptomic shifts consistent with a growth–defence trade-off under heat–drought stress [38]. In the present study, persimmon trees responded positively to calcium pre-anthesis treatments, improving fruit set and reducing premature fruit drop (Figure 1A) caused by warm summer conditions.

But how does an element such as Ca inhibit the abscission process? Abscission is a tightly regulated process integrating environmental, hormonal, metabolomic, and genetic cues [6], yet its molecular regulation remains uncharacterised in persimmon. During the first physiological fruit drop, persimmon fruit abscission follows a conserved molecular programme previously described in *Arabidopsis thaliana* [39] and in woody species such as citrus and mango [16,40]. Reduced auxin transport, mediated by the efflux carrier *DkPIN1*, promoted ethylene biosynthesis, which subsequently induced *DkIDL6* expression and triggered the transcriptional cascade of stage C abscission-related genes [41]. This regulatory sequence culminated in the activation of PG, driving cell wall pectin degradation within the AZ (Figure 2A–D).

The intense first fruit drop observed (Figure 1A) reflects the excessive spring flowering characteristic of persimmon, with the tree ultimately retaining only those fruits that can be sustained by available resources [42]. The balance between carbohydrate supply and demand plays an important regulatory role in fruit shedding [43]. Consistent with observations in citrus, where reduced CH availability accelerates abscission [44,45], we detected lower CH levels in abscised fruits (Figure 3A). This agrees with source–sink ratios since reports in apple and citrus show that lower source–sink balance or weaker fruit sinks increase early fruitlet drop [46,47]. The lower sugars in abscising fruitlets can be explained by two non-exclusive processes. First, fruitlets destined to abscise stop growing earlier, losing sink activity and import capacity. This is supported by transcriptomic and growth data showing that such fruitlets had ceased development before dropping, consistent with a curtailed sugar supply [48]. Second, abscising fruitlets appear more stressed, which raises respiratory demand and sucrose cleavage via invertases and sucrose synthase, thereby depleting hexoses prior to shedding. Under conditions of carbon starvation or water/heat stress, fruit sugar pools decline before the abscission peak, evidencing enhanced consumption at the sink [49]. Mechanistically, glucose is funnelled preferentially into central metabolism because it is rapidly phosphorylated by hexokinase (HXK), a key metabolic and stress-signalling node. In contrast, fructose relies mainly on fructokinase (FRK), and limited FRK capacity under stress can cause relative fructose retention while glucose is depleted faster, matching the Glc < Fru pattern observed in dropped fruitlets [50,51]. In parallel, stress-integration pathways (SnRK1/HXK/TOR) coordinate catabolic up-regulation under energy deficit, further biasing hexose uses towards ATP-producing routes [52]. Although the SnRK complex was activated, triggering the nutritional deficit signalling pathway, carbohydrate homeostasis could not be restored. Starch hydrolysis, a canonical mechanism for remobilising sugars under stress [53], was induced but failed to generate sufficient substrates to sustain the activity of key sucrose metabolism enzymes (Figure 3A–E). In the second wave of fruit drop, the drop related to weather abiotic stress conditions, untreated trees displayed the same abscission mechanism as in the first wave, confirming its recurrence in persimmon. Interestingly, at this stage, Ca was able to modify the process (Figure 4) and prevent premature fruit abscission (Figure 1A). These results are consistent with previous studies since field evidence indicates that preharvest Ca applications can reduce fruit drop across woody fruit crops. It has been reported that foliar CaCl_2_ lowered premature drop by 23–81% in highbush blueberry, and even in the ‘Costata’ persimmon variety, calcium sprays (with/without girdling) significantly reduced June and preharvest drop [54,55]. In the present study, treated trees displayed higher Ca concentrations in the calyx compared with controls (Figure 4A). Retained calyces from Ca-treated trees accumulated more Ca than those from controls, although abscised fruits showed even higher concentrations than retained ones, likely due to a biomass dilution effect [56]. A similar trend has been observed in Siberian apricot [57]. In contrast to abscising fruitlets, retained fruit continued to develop, expanding biomass and importing assimilates. As a result, the fraction of non-calcium solids (carbohydrates, newly deposited cell wall polymers, proteins, and other nutrients) increased, diluting the apparent Ca concentration despite ongoing Ca delivery, a classical biomass-dilution effect documented across fruits and vegetables [58]. In parallel, the transpiration in less developed fruits during stress periods (Figure 1C,D) is typically higher than in fully developed ones [59], promoting greater Ca mobilisation towards these growing organs [60]. Both the concentration levels of Ca and its form in the cellular medium can affect fruit physiology: the increase in Ca in its ionic form within the cytosol can act as an abscission signal [10], and an excess of extracellular Ca may promote the ectopic deposition of calcium oxalate crystals [61]. Nevertheless, optimal Ca concentrations (1–5 mM) play a key role in protecting the cell wall integrity from ruptures associated with senescence [62].

Regarding the relative expression of genes, the results of this study indicate that exogenous Ca dampens the abscission program at the transcriptional level. This is evidenced by the overall reduction in *DkIDL6* and the enzymes *DkPG20* and *DkPME41* in both retained and dropped fruits, as well as by the absence of a compensatory increase in abscission markers despite the lower *DkPIN1* signal in retained fruits. In this sense, reducing the auxin-export signal (*DkPIN1*) in retained fruit did not translate into activation of the abscission cascade because Ca concurrently suppressed the core effectors of cell separation (IDA/IDL–PG/PME axis), leading to greater cell wall cohesion and enhanced fruit firmness at harvest. This is consistent with reports in other fleshy fruit where Ca treatments down-regulate cell wall hydrolase genes and activities and stabilise wall structure/firmness [63,64,65]. Mechanistically, this fits the broader view that Ca modulates AZ metabolism and cell wall integrity, thereby lowering abscission propensity and increasing fruit retention. These results are consistent with those of [66], who reported that Ca-treated fruits exhibited a denser middle lamella and increased cell adhesion. Conversely, Ca removal from cells using chelating agents rapidly activates PG-mediated cell wall degradation, resulting in organ abscission [27,67].

In addition, exogenous Ca applications have been shown to improve abiotic stress tolerance in more than 15 species [68]. During periods of water stress (Figure 1D), Ca has been reported to stabilise chloroplast structure, maintaining normal photosynthetic function [69], and to reduce stomatal aperture, facilitating adaptation to drought conditions [70]. Altogether, these effects can alleviate plant stress and reduce the expression of the abscission activator *DkIDL6* (Figure 4B).

Overall, these results suggest that the abscission mechanism in persimmon fruits is similar to that described in the model plant *Arabidopsis thaliana* and that Ca applications can reduce premature fruit abscission under environmental stress by decreasing the upstream expression of *DkIDL6* at the onset of stage C and by limiting the activity of the hydrolytic enzymes PG and PME during the final phases of cell wall degradation.

In conclusion, under Mediterranean heat–drought conditions, four consecutive Ca applications (2.5 cm^3^ L^−1^ every 15 days starting at pre-anthesis) reduced premature fruit drop in persimmon by 30%. This effect was associated with the attenuation of the abscission programme at the transcriptional level by the reduced expression of *DkIDL6*, *DkPG20*, and *DkPME41*, while preserving cell wall cohesion and firmness. The canonical auxin-to-ethylene cascade operated during the first drop, but Ca prevented its re-engagement in the second wave despite a lower *DkPIN1* signal in retained fruit, indicating that suppressing the IDL–PG/PME axis overrides any auxin-export reduction. Apparent higher Ca concentrations in the dropped fruit reflect growth arrest and dilution differences, rather than greater Ca uptake. Together with the stress-mitigating effects of Ca (improved photosynthetic integrity and tighter stomata control), these mechanisms reduce abscission propensity and increase fruit retention. Practically, these findings support timely Ca spraying (pre-anthesis/early fruit set) as a climate-smart tool to stabilise persimmon yield while considering species/cultivar differences, timing dependence, and the need to optimise dose and formulation.

## 4. Materials and Methods

### 4.1. Plant Material and Experimental Layout

The experiment was conducted in a commercial plantation of 15-year-old ‘Rojo Brillante’ persimmon trees grafted onto *Diospyros lotus* rootstock, planted at a spacing of 5 x 4 m, fertilised, drip irrigated, and managed according to standard agronomical practices. The experimental field was in Montaverner (València), Spain (38°53′ N, 0°28′ W).

Calcium application (BARRIER^®^, Ca 20.7% + SiO_2_ 24%, Cosmocel Ibérica S.L) was studied in 15 trees, divided into three treated groups of five trees each. Fifteen untreated control trees were arranged similarly. Treatments were applied just before anthesis (5 April) at a concentration of 2.5 cm^3^ L^−1^, sprayed by a Backpack sprayer at a rate of 3 L tree^−1^ (tree volume of 12 m^3^). Applications were repeated every 15 days until 5 June, for a total of four splits.

### 4.2. Field Measurements

Nets were placed under the trees to collect and count abscised fruits. To ensure that fruit drop corresponded precisely to each sampling date, all fallen fruits were removed from the nets the day before sampling. At each sampling time (approximately every 15 days), newly abscised fruits were counted and removed, while fruits retained on the trees were sampled directly. Temperature and rainfall were recorded using the RED SIAR-IVIA climatological station located in Bèlgida, close to the field site. Final yield was assessed in October. In addition, fruit quality parameters were measured on 50 fruits per treatment (control and calcium applications), including weight (analytical balance; Sartorius, Göttingen, Germany), diameter (digital calliper; Mitutoyo, Kawasaki, Japan), colour (Minolta Colorimeter, Model CR-300; Ramsey, NY, USA), and firmness (Texturometer Model 4301; Instron Corp., Canton, MA, USA).

### 4.3. Gene Expression Analysis

The abscission mechanism was investigated by analysing the expression of key regulatory genes across the different stages of AZ degradation. Twelve retained and twelve dropped fruits were sampled from control trees during the first physiological fruit drop (5 May) to generate a large population for defining the persimmon abscission mechanism. During the second wave (31 May), five retained and five dropped fruits per treatment were sampled to assess the effect of Ca on the underlying mechanisms. Samples were immediately frozen in liquid nitrogen. At stage B, the expression of *PIN-FORMED1* (*PIN1*), an auxin efflux carrier, was measured to evaluate polar auxin transport [71]. At stage C, following Estornell et al. (2013) [6], the expression of *INFLORESCENCE DEFICIENT IN ABSCISSION* (*IDA/IDL6*), the gene that triggers abscission, was examined. Finally, the expression of *POLYGALACTURONASE20* (*PG20*) and *PECTIN-METHYLESTERASE41* (*PME41*) was assessed to characterise cell wall degradation at the final phase of stage C [16]. The expression of these genes was studied in the AZ tissues.

During the first physiological fruit drop, associated with carbohydrate starvation from the high number of developing organs [47], sugar metabolism and starch hydrolysis were investigated in the flesh of whole fruit after peel removal by analysing the expression of *CELL WALL INVERTASE* (*CWIN*), *CYTOPLASMIC INVERTASE* (*CIN*), *VACUOLAR INVERTASE* (*VIN*), and *α-AMYLASE* (*α-AMY1*), as described by Ruan et al. (2014) [53] and Smith et al. (2005) [72]. In addition, the expression of *SUC NON-FERMENTING-RELATED KINASE 1* (*α-SnRK1*), a gene directly associated with the activation of signalling pathways under carbohydrate deficiency [73], was also measured.

Within the *PIN*, *PG*, and *IDA/IDL* gene families, there is a large number of paralogues with potential roles in abscission. Therefore, the selection of the genes analysed in this study is based on previously published evidence from woody species. *PIN1* is widely associated with polar auxin transport in citrus and apple [74,75], while *PG20* shows the highest activity in the abscission zone of citrus among all *PG* genes evaluated to date [16]. Regarding the *IDA/IDL* family, its direct involvement in organ abscission in mango, citrus, and litchi is well documented [6,40,76]. Within this family, the paralogue *IDL6* is selected due to its close association with calcium signalling in the abscission zone [77].

Total RNA was isolated following the protocol described by Martínez-Fuentes et al. (2014) [78] for loquat (Eriobotrya japonica), a recalcitrant species similar to persimmon. All procedures were performed on ice, with centrifugation steps at 4 °C, and all reagents were obtained from Sigma-Aldrich^®^ (Merk Group, St. Louis, MO, USA). RNA quality was assessed using a Nanodrop ND-1000 spectrophotometer by measuring the OD260/OD280 ratio and further confirmed by gel electrophoresis. Transcripts from 1 μg of total RNA were reverse-transcribed using the *PrimeScript™ RT Reagent Kit* (Perfect Real Time; TAKARA Bio Europe, Saint-Germain-en-Laye, France) in a final reaction volume of 10 μL. For each amplification reaction, 2 μL of four-fold-diluted first-strand cDNA was used as a template. Real-time quantitative PCR (RT-qPCR) was performed on a *Rotor-Gene Q 5-Plex system* (Qiagen, Germantown, MD, USA) with the *TB Green^®^ Premix Ex Taq™ PCR Kit* (TAKARA Bio Europe, Saint-Germain-en-Laye, France). The amplification protocol consisted of an initial pre-incubation at 95 °C for 15 min, followed by 40 cycles of denaturation at 94 °C for 15 s, annealing at 60 °C for 30 s, and extension at 72 °C for 30 s. Each RT-qPCR reaction was performed in triplicate per gene, with fluorescence monitored in real time using the Rotor-Gene detector. Relative transcript abundance was determined from threshold cycle (Ct) values using the 2-ΔΔCt method [79]. Gene expression levels were normalised against the reference gene *β-LYC* (*β-lycopene cyclase*), whose expression is linked to fruit colour changes but remains stable in the studied tissue [80]. For each experiment, the lowest expression value among samples was used as the baseline for normalisation. Three independent biological replicates were analysed under each experimental condition, with three technical replicates per sample.

### 4.4. Sequence Analysis

Based on previous studies in woody species, tomato, and Arabidopsis [6,16,53,71,81,82,83], the sequences of the genes of interest were identified using BLAST searches against the PHYTOZOME v.14 (https://phytozome-next.jgi.doe.gov/, accessed on 5 May 2025) and PERSIMMON DB genome (https://persimmon.kazusa.or.jp/, accessed on 5 May 2025) databases. Multiple sequence alignment and phylogenetic analysis were carried out using MEGA11 software (https://www.megasoftware.net/, accessed on 15 May 2025). The genes included in this study are orthologues of *Arabidopsis thaliana*, and primers were designed using Primer 3 v4.1.0 (http://primer3.ut.ee/, accessed on 16 May 2025). Primer sequences are provided in Supplementary Table S1. 

### 4.5. Ethylene Determinations

Ethylene production was measured in retained and dropped fruits during physiological fruit drop (5 May). Three replicates of three fruits were enclosed in 1.5 L hermetic jars with a 1.5 cm diameter rubber stopper (septum). After 24 h of incubation at 20 °C, a 1 mL gas sample was withdrawn from the headspace of the container and injected into a gas chromatograph (Perkin Elmer, model: 2000, Norwalk, CT, USA), equipped with a propack QS 80/100 column and a flame ionisation detector.

### 4.6. Carbohydrate Analyses

Powdered samples (100 mg DW) were prepared from the flesh of 20 retained and 20 abscised fruits (after peel removal) collected during physiological fruit drop (5 May). Plant material was oven-dried at 65 °C to constant weight and ground into a fine powder (<0.5 mm) prior to carbohydrate extraction, as commonly applied for sugar determination in plant tissues [84]. For carbohydrate analysis, 0.4 g of fruit pulp (dried weight) was mixed with 1.3 mL of distilled water in an Eppendorf tube and vortexed for complete homogenization. The addition of distilled water was considered in the dilution factor for subsequent calculations. Samples were centrifuged for 20–30 min at 12,000 rpm (Eppendorf 5810R centrifuge, Hamburg, Germany), and the supernatant was collected, filtered through a 0.45 µm nylon filter, and stored at −20 °C until analysis. Sugars were analysed by HPLC (Waters 515 pump, Waters 2414 refractive index detector) equipped with a 5-μm Tracer Carbohydrate column (250 mm × 4.5 mm; Teknokroma, Barcelona, Spain). The mobile phase consisted of acetonitrile–water (75:25, *v*/*v*) at a flow rate of 1 mL min^−1^. Fructose, glucose, and sucrose were identified by comparison with analytical standards (Sigma, Barcelona, Spain) and quantified using external calibration curves. Extraction efficiency was checked using fucose as an internal standard, absent in the natural samples. Concentrations obtained from the chromatographic system (in mg mL^−1^) were expressed as mg g^−1^ dry weight according to the relationship

$$\mathrm{mg}\;g^{-1}=\frac{measured\;concentration\;\left(\mathrm{mg}{\mathrm{mL}}^{-1}\right)}{sample\;weight\;(g)\;\times 1.3\mathrm{mL}}$$

### 4.7. Calcium Analysis

Calcium concentration was determined using inductively coupled plasma atomic emission spectrometry (ICAP-AES 6000, Thermo Scientific, Cambridge, UK) following nitric–perchloric acid digestion [85]. Dried plant material (0.5 g) was predigested overnight with 10 mL of HNO_3_ on a digestion block at 120 °C. After cooling the samples to room temperature, 2.0 mL of 70% ultratrace-metal grade HClO_4_ was added, and the mixture was redigested at 220 °C until the appearance of white fumes. The digested samples were then diluted to a final volume of 25 mL with ultrapure water, and Ca concentrations were subsequently measured by ICAP-AES [86].

### 4.8. Statistical Analysis

Analysis of variance was carried out using Student’s *t*-test to separate means at significance levels of *p* ≤ 0.01 and *p* ≤ 0.05, with *Statgraphics Centurion XVI* software (Statistical Graphics, Englewood Cliffs, NJ, USA). Percentage data were analysed following an arc-sine √p transformation.

## Figures and Tables

**Table 1:** Effect of calcium treatments on fruit characteristics at harvest (fruit weight, diameter, height, colour, and firmness were measured for 50 fruits per treatment). Different letters indicate statistically significant differences (*p* ≤ 0.05).

Treatment	Weight (g)	Diameter (mm)	Height (mm)	Colour (1000 a/Lb)	Firmness (N)
Control	216.76 b	74.0 a	72.7 b	7.32 a	39.5 b
Ca	239.79 a	74.4 a	76.4 a	6.26 b	43.5 a

## Data Availability

The data are available from the authors upon request.

## Supplementary Materials

The following supporting information can be downloaded at https://www.mdpi.com/article/10.3390/plants14223482/s1, Table S1: Primer sequences used in this study.
