# Supplementary material for: Calcium Reduces Fruit Abscission in Persimmon by Targeting Cell Wall Integrity
Source: Plants (Basel). 2025 Nov 14;14(22):3482. doi: 10.3390/plants14223482 (PMC12655806; doi:10.3390/plants14223482)
Supplement: Supplementary file 1 [file plants-14-03482-s001.zip › plants-3970049-supplementary.pdf]

Supplementary Table S1. Primer sequences used in this study

Gene	Persimmon ID	Arabidopsis Orthologe	Description	Forward primer (5'-3')<br>Reverse primer (5'-3')
DkPIN1	DKAch03a26465.t1	AT1G73590	Auxin efflux carrier	CGAGTATAGAGGCGCCAAGA<br>TGACGTGAAGCTTCCCATCT
DkIDL6	DKAch02a09592.t1	AT5605300	Inflorescence deficient in abscission-like	TGGCCAAATTTGTCGACCTC<br>GATCCATATTTTCCGGCGGG
Dkα-SnRK1	DKAch15y11961.t1	AT3G01090	Serine threonine kinase (subunit α)	TGAGATTCGTCAGCACCCTT<br>CCCTCATTCTGTACTCGGCT
Dkα-AMY1	DKAch05a16351.t1	AT4G25000	Alpha-amylase	AATCAGGGCAAGGAATGGGA<br>TTTCTCCCACACGGCATAGT
DkCWIN	DKAch03a23642.t1	AT3G13790	Cell wall Invertase / Sucrose enzyme	CTTGTGATCGTCAGCCATGG<br>CTCGAACAGACGGCTTGATG
DkCIN	DKAch15y10840.t1	AT4G09510	Cytosolic Invertase / Sucrose enzyme	GCCGATGGTTGCTCAATGAT<br>ACTCCTCCGTTTTGTAGCGA
DkVIN	DKAch01a03133.t1	AT1G62660	Vacuolar Invertase / Sucrose enzyme	CAACCTATCCGATCCCCTCC<br>CATGCATAACCCCGTCCAAG
DkPG20	DKAch12a27112.t1	AT3G07970	Polygalacturonase	GGCCAAGCTTTCAGGAACAA<br>GGCATGGCTTCTCTTGATCG
DkPME41	DKAch09a30910.t1	AT3G59850	Pectin-methylesterase	TCTTGATCAGTGGGTTGGCA<br>CATTGGAGTTGAGGATGGCG
DkLYC	DKAch09a29136.t1	AT3G10230	Reference gene	GCTTCCCTTGTATGACCCCT<br>ACTCATCCACCCAAACACCA
